# Self-Powered Deep-Ultraviolet Photodetector Driven by Combined Piezoelectric/Ferroelectric Effects

**DOI:** 10.3390/nano14231903

**Published:** 2024-11-27

**Authors:** Vo Pham Hoang Huy, Chung Wung Bark

**Affiliations:** 1 Department of Electrical Engineering, Gachon University, Seongnam 13120, Republic of Korea; vophamhoanghuy@yahoo.com.vn; 2 Department of Semiconductor Engineering, Gachon University, Seongnam 13120, Republic of Korea

**Keywords:** ultraviolet C (UVC) photodetector, β-phase polyvinylidene fluoride, β-phase gallium oxide, ferroelectric effect, self-powered

## Abstract

In this study, in situ piezoelectricity was incorporated into the photoactive region to prepare a self-powered deep-ultraviolet photodetector based on a mixture of polyvinylidene fluoride (PVDF)@Ga_2_O_3_ and polyethyleneimine (PEI)/carbon quantum dots (CQDs). A ferroelectric composite layer was prepared using β-Ga_2_O_3_ as a filler, and the β-phase of PVDF was used as the polymer matrix. The strong piezoelectricity of β-PVDF can facilitate the separation and transport of photogenerated carriers in the depletion region and significantly reduce the dark current when the device is biased with an external bias, resulting in a high on/off ratio and high detection capability. The self-powered PD exhibited specific detectivity (D* = 3.5 × 10^10^ Jones), an on/off ratio of 2.7, and a response speed of 0.11/0.33 s. Furthermore, the prepared PD exhibits excellent photoresponse stability under continuous UV light, with the photocurrent retaining 83% of its initial value after about 500 s of irradiation. Our findings suggest a new approach for developing cost-effective UV PDs for optoelectronic applications in related fields.

## 1. Introduction

Photodetectors are frequently engaged in electro-optics, biomedicine, communications, security, and various related fields. As high-tech applications such as optical storage, lidar, and communication emerge, photodetectors essentially satisfy ever-tougher specifications [1,2,3]. Because of their significant value in military and commercial applications such as flame detection, missile warning, biological investigation, etc., PD devices that operate in the deep-ultraviolet (UVC) band have garnered great attention [4,5,6]. Specifically, it is necessary to reduce the power consumption of UVC detectors, which should ideally be 0 V, in addition to creating devices with high sensitivity, quick reaction times, and most importantly, solar-blindness (i.e., the capacity to be immune to visible, infrared, and UV rays above 280 nm). The lack of UVC fluence meters appropriate for UV decontamination procedures of personal protective equipment (e.g., face shields, surgical masks) in biosafety cabinets, a common feature of many academic, public health, and hospital laboratories, might be made up for by a self-powered UVC detector, since it would be portable, small, and easy to use [7]. Girolami et al. prepared a self-powered solar-blind UVC detector by combining the idea of asymmetrical area Schottky connections to diamonds with a unique “sandwich (i.e., vertical) construction. The PD device demonstrated an ultraviolet-to-visible (UV/Vis) rejection ratio of 6.8 × 10^3^ under photovoltaic operating conditions, thanks to a straightforward fabrication process, the asymmetry of the metal contacts, and the exceptional charge transport characteristics of the diamond active medium, which had the highest mobility lifetime (μτ) of all documented photogenerated carriers. Furthermore, when bias was applied, the UV/Vis rejection ratio achieved an outstanding value (>10^4^) even at very low electric fields (0.01 V/μm) [8]. TiO_2_ nanoparticles with acetate groups on the surface were created when Hoang et al. used acetic acid (HAc) to modify TiO_2_ particles to create promising optical materials. According to the study, adding the right quantity of acetate functional groups to TiO_2_ nanoparticles greatly decreased the O vacancies and increased the number of energy states in the TiO_2_ energy band. The post-treatment with HAc inhibited the undesired absorption of visible light by TiO_2_ and expanded its bandgap to 3.83 eV. Consequently, under 254 nm UV light, the constructed device showed a high specificity of 3.38 × 10^11^ Jones and a quick response time of 64/106 ms (rise/fall time), allowing for self-powered operation [9]. Huyen et al. tuned the ideal transparency in the UVC region by fabricating ITO thin films with different sputtering periods using the magnetron sputtering process. Particle transfer from the target to the substrate was constant as a result of the plasma bombardment of the target material. As a result, the produced ITO films demonstrated consistent capacity for commercial manufacturing in addition to exceptional optoelectronic performance. In this study, an ITO thin film with a thickness of 26.6 nm demonstrated strong conductivity (11.3 S/cm) for charge transfer in PD and remarkable optical transmittance in the deep-UVC region (67% at 254 nm). Under 254 nm UVC irradiation, the perovskite PD employing the produced ITO-coated quartz substrate demonstrated exceptional performance at zero bias with an on/off ratio > 104 and a sensitivity of 250 mA/W [10]. Zhou et al. investigated high-performance self-powered DUV PDs based on MoS_2_/GaN p-n heterojunction. Beyond the limitations of lattice matching, a good heterojunction can arise because of the free dangling bonds on the MoS_2_ film’s surfaces. Once the p-n heterojunction between the MoS_2_ film and the GaN substrate forms, electrons in the MoS_2_ film will tend to migrate to the GaN side, whereas holes in the GaN will migrate to the MoS_2_ film due to the difference in Fermi level (EF). Absorption of the incident light under UV light illumination produces electron–hole pairs, which are then swiftly separated by the internal electric field and transported to the electrodes, resulting in photocurrent and a speedy reaction time. At zero bias voltage, this PD has quick response times of 46.4/114.1 μs (5 kHz), a high specific detectivity of 2.34 × 10^13^ Jones, and a high responsivity of 187 mA W^−1^ [11].

During recent decades, the substitution of organic elements in a range of electronic devices with inorganic components has been widely promoted [12,13,14,15,16,17,18,19]. The numerous benefits of employing organic materials, including their light weight, flexibility, affordability, solution processability, and ease of large-scale fabrication, further promoted these initiatives [20,21,22]. Such a remarkable quality of organic compounds encourages the simplification of the spectrally selective fabrication method for photodetectors (PDs), which lowers the cost of manufacturing. In addition, their mechanical flexibility makes wearable PDs feasible, which has enormous potential for employment in health detection devices. In contrast to inorganic PDs (IPDs), organic PDs (OPDs) have a comparatively slower response time (10^−3^–10^−1^ mA W^−1^) and less carrier generation due to limited carrier mobility (10^−5^–10^−4^ cm^2^·V^−1^·s^−1^) and disordered molecular alignment of organic molecules [12,23]. Consequently, assorted engineering has been utilized to reinforce the performance of PDs, comprising the development of donor and acceptor materials with suitable molecular structures [24,25,26], the modification of interface layers [27,28], and the incorporation of additives [29,30]. Remarkably, an efficient and straightforward approach for increasing photocurrents, independent of OPD structure, requires meticulously selecting and refining the electron transport layer (ETL) process [31,32,33,34]. Ga_2_O_3_-based materials have attracted significant attention because of optical communication and their potential utilization in imaging, fire monitoring, and high-voltage corona sensing [35,36,37,38,39,40]. Particularly, Ga_2_O_3_ has a high bandgap energy (4.7–5.4 eV), making it exemplary for visible–blind sensors. Furthermore, its five crystal phases (ε, δ, γ, β, and α) promote the selective use of the Ga_2_O_3_ crystal phase according to specific optical and electrical requirements [41,42]. Since the other phases are metastable, the β-phase of Ga_2_O_3_ has been the object of most research efforts due to being stable [43]. Furthermore, β-phase Ga_2_O_3_ has been demonstrated to have promising potential as a material for next-generation solar-blind PDs due to its exceptional chemical and thermal stability, high breakdown electric field (8 MV/cm), and suitable bandgap (4.9 eV) [44,45]. Among these phases, β-Ga_2_O_3_ has been determined to be a feasible phase for solar-blind sensors without the need for a bandgap technique because of its intrinsic solar-blindness and appropriate bandgap of around 4.8 eV. Its thermal and chemical stability and superior mechanical characteristics also make it a suitable replacement for the utilization of solar-blind PDs. Solar-blind PDs based on Ga_2_O_3_ in bulk single crystals, nanostructures, polycrystalline films, epitaxial thin films, and even amorphous layers have achieved significant strides so far [46,47,48,49,50,51,52,53]. For instance, a polycrystalline β-Ga_2_O_3_ thin-film PD developed on an inexpensive Si substrate was investigated by K. Arora et al. Under the influence of monochromatic light at a wavelength of 250 nm, the PD device generates an external quantum efficiency (EQE) of 4.80 × 10^4^% at 5.0 V and a responsivity of 96 × 10^3^ mA W^−1^. Additionally, the designed photodetector indicates a great ability to detect weak incident signals at the deep-UV area (43 nW cm^−2^) [54]. According to Qian et al., the PD device made from amorphous Ga_2_O_3_ films sputtered by radiofrequency magnetron has a high responsivity (70 × 10^3^ mA W^−1^) and large specific detectivity (1.26 × 10^14^ Jones) [55]. Consequently, high-performance PD devices are not confined by the feature of Ga_2_O_3_ films. It has been reported that polycrystalline Ga_2_O_3_ films may be manufactured using a straightforward procedure in addition to epitaxy or deposition techniques, namely GaN thin-film oxidation at high temperatures [56]. According to Weng et al., UV PD devices manufactured from furnace-oxidized Ga_2_O_3_ thin films have a responsivity of 0.453 A W^−1^. Nonetheless, the performance of commercial sensors currently on the market is far from such low responsivity, particularly when it comes to the detection of very weak signals. Unfortunately, extended photocurrent build-up and decay durations on the scale of many seconds are frequently associated with the external quantum efficiency high gain, which poses a challenge for real-world applications. Therefore, in order to build a high-performance PD device, additional technological advancements in the growth of the β-Ga_2_O_3_ absorber layer and device manufacturing are still needed.

In this study, we designed a sensitive deep-solar-blind photodetector based on a polymer blend with semiconducting β-Ga_2_O_3_ nanorods to form an efficient layer. This layer acts as an active layer, promoting energy barrier transformation via the photo-gating effect; the trapped carriers at the interface of organic photoactive materials, namely carbon-quantum dots (CQDs), are more efficiently extracted towards the electrode instead of recombination at the PEI/CQD interface. Furthermore, our design takes into account an appropriate structure with straightforward solution procedures at low temperatures. This device demonstrates performance for a self-powered 254 nm photodetector compared to previous research, with a detectivity of 2.4 × 10^10^ Jones and sensitivity of 3.72 mA W^−1^ under 254 nm radiation with an irradiance power of 1.14 mW cm^−2^ at zero bias. The introduced piezoelectric property also contributes to the versatile usability of this PD device. 

Recently, electro-optic devices have felt the consequences of the application of piezoelectric materials, which has opened up previously unheard-of possibilities for device applications [57,58,59]. β-Poly(vinylidene fluoride) (β-PVDF) has been regarded as the perfect material for detector applications because of its high solubility, outstanding piezoelectricity, and ease of processing [60,61,62]. Our hybrid interconnected photodetector first achieves ferroelectric behavior when biased at +10 V, due to the ferroelectricity of PVDF, when the PD device is operated on CQDs under UVC illumination. An internal electric field is generated, and exciton dissociation is assisted by the continuous polarization of the ferroelectric PVDF. In addition to providing the piezoelectric impact, the β-PVDF layer can act as a flexible substrate, which can hinder the mobility of injected electrons via the trapping site in PEI/CQDs, restrict recombination with the photogenerated hole, and create a photogenerated electron transport channel through ß-Ga_2_O_3_, enabling efficient transport carriers. The device, after positive piezoelectricity, provides performance with a detectivity of 3.5 × 10^10^ Jones and a responsivity of 0.90 mA W^−1^. Multiple conventional separate detectors can be combined into a single device using this type of multi-functional device to provide the same system performance in a very compact device footprint, or, in other words, create a new device with multiple uses. Thus, the results provide a viable approach for improving the performance of photodetectors for industrial applications. This work provides a feasible solution to create new types of UVC PDs, with economic efficiency and high applicability, because of the following characteristics: (i) the design of the device considers a structure suitable for simple solution processes at low temperatures (60 °C); (ii) β-PVDF@β-Ga_2_O_3_ promotes the ferroelectric properties of the organic-based UVC device for the first time; (iii) the ferroelectric layer promotes the efficient extraction of the trapped charge carriers at the interface; and (iv) the β-PVDF layer can hinder the mobility of injected electrons via the trapping site in PEI/CQDs

## 2. Experimental Section

### 2.1. Materials

D-glucose (99.5%), sodium hydroxide (97%), and magnesium sulfate heptahydrate (≥99%) were used to prepare photoactive CQDs. β-Ga_2_O_3_ was prepared using gallium (III) nitrate (Ga(NO_3_)_3_, 99.9%) as a precursor. The polymer used in this study, polyvinylidene fluoride (PVDF; Mw ≈ 534,000), was dissolved in dimethylacetamide (DMAc; anhydrous, 99.8%), while polyethylenimine (PEI; branched, Mw = 25,000) was dissolved in 2-propanol (IPA; 99.5%). The hole transport layer poly(3,4-ethylenedioxythiophene)-poly(styrenesulfonate) (PEDOT:PSS; Orgacon dry redispersible pellets) was dissolved in dimethylacetamide (DMAc; anhydrous, 99.8%) with DMSO (anhydrous, >99.9%). The remaining solvents, such as ethyl alcohol (anhydrous, 99.9%), distilled water, and acetone (≥99.5%), were used to wash the FTO surface. All chemicals were supplied by Sigma-Aldrich in Seoul, Korea. The details of the preparation of Ga_2_O_3_ and the PD device are presented in Section 2.2 and Section 2.3.

### 2.2. Synthesis of β-Ga_2_O_3_

A simple method based on an ultrasonic chemical procedure was utilized to synthesize ß-Ga_2_O_3_ [63]. In a round-bottomed flask with a stirring paddle, 0.1 mol/L of gallium nitrate aqueous solution was added. Then, the round flask containing the compound was submerged in a 95 °C water bath, and a grating was introduced from a specific quantity of ammonia solution to adjust the pH = 9. After 5 h of heating, a white precipitate was formed. In the final step, the desired Ga_2_O_3_ nanorods were obtained by calcining the white precipitate for 120 min at 700 °C.

### 2.3. Fabrication of Photodetector Device

**Figure 1:**
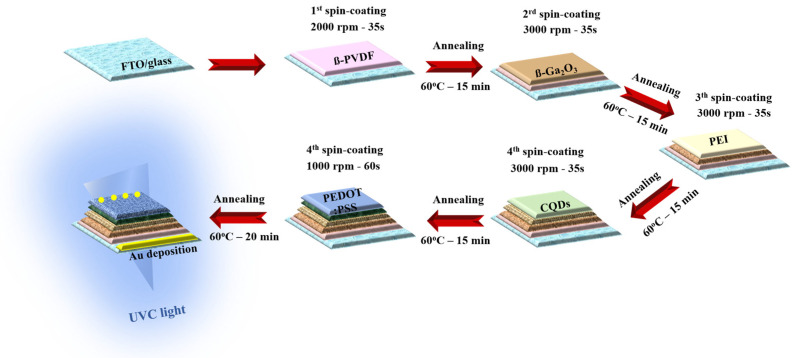
The schematic of UV PD preparation.

The PD device was prepared according to a vertical architecture, employing an uncomplicated solution procedure. First, the FTO-coated glass surface was completely purged in an ultrasonic bath every ten minutes using acetone, IPA, DI water, and ethanol, in this order. The piezoelectric layer was prepared by forming ß-PVDF and ß-Ga_2_O_3_. PVDF was spin-coated onto FTO-coated glass (with concentrations of 10, 50, 100, and 150 mg/mL in DMAc) at 2000 rpm for 35 s. Then, Ga_2_O_3_ was spin-coated onto the PVDF layer (with contents of 25, 20, 15, 10, and 5 mg mL^−1^ in IPA) at 3000 rpm for 30 s. Next, the UVC absorber layer was formed by successively coating layers of PEI (7 mg/mL in IPA) and CQDs (diluted concentration in ethanol solution at 30%) at 3000 rpm for 30 s, during which CQDs were used according to the study of Li et al. [64]. On the surface of CQDs, the top electrode was formed. PEDOT:PSS at a 1.3 wt% concentration in an IPA solution with 6 wt% assisting DMSO was spin-coated for 60 s at 1000 rpm. All layers were calcined on hot plate for 15 min at 60 °C. The uppermost layer was then deposited by thermally evaporating 100 nm of Au on top of the apparatus at a high vacuum pressure of 8 × 10^6^ Torr. The schematic below depicts the PD device process (Figure 1).

### 2.4. Characterization and Measurement

Scanning electron microscopy (SEM, Hitachi SU8600, Tokyo, Japan) and X-ray diffraction (XRD, SmartLab, Rigaku, Tokyo, Japan) were utilized to determine the crystallographic and morphological characteristics of the as-prepared ß-Ga_2_O_3_. The bandgap and optical properties were analyzed using a UV–visible (UV–Vis) spectrometer (LAMBDA 750, Perkin Elmer, Waltham, MA, USA). The excitation wavelength of 260 nm was determined by photoluminescence (PL, Fliorolog–QM, Horiba, Kyoto, Japan). The separation of layers of ß-Ga_2_O_3_, ß-PVDF, and the PD device was demonstrated via cross-sectional SEM. The electrical properties of the as-fabricated photodetector were assessed using the current–time (I-t) and current–voltage (I–V) profiles using a source meter (Keithley 2400, Tektronix, Beaverton, OR, USA) under UV light with wavelengths of 254, 302, and 365 nm (Spectroline UV5NF, Spectronics Corporation, Melville, NY, USA). A potentiostat–galvanostat (Bio-Logic SP−240) was used to examine the potential. To ascertain the photoresponse, a 254 nm UV lamp (Vilber Lourmat VL-6.LC) was used. A Keithley source meter (2400) was used to record the electrical signal.

## 3. Results

**Figure 2:**
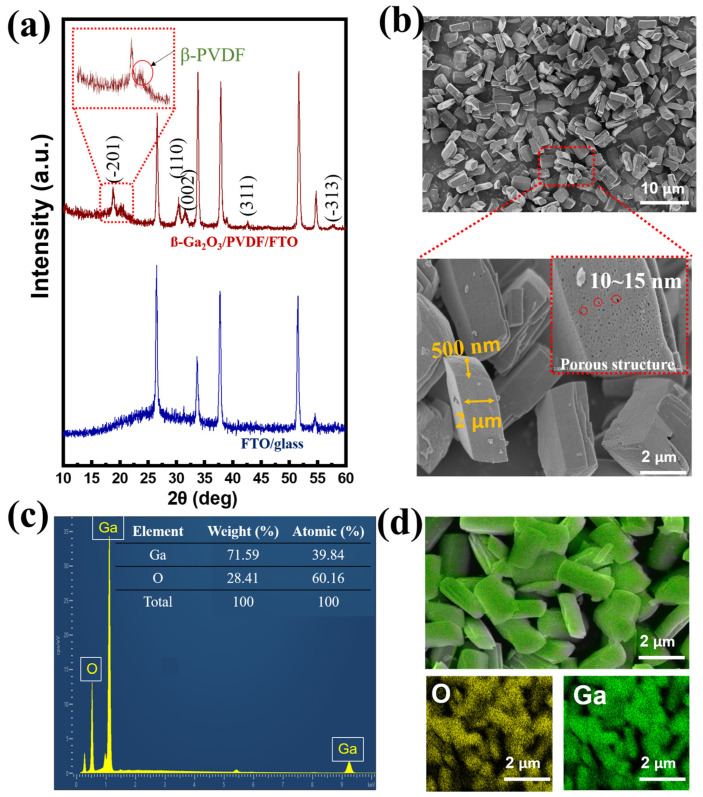
(**a**) X-ray diffraction patterns of FTO/glass substrate and ß-PVDF@ß-Ga_2_O_3_-FTO, (**b**) SEM image, (**c**) EDureX analysis, and (**d**) elemental mapping of as-prepared β-Ga_2_O_3_.

Using scanning electron microscopy (SEM) and X-ray diffraction (XRD), the characteristics of the piezoelectric layer were examined. The presence of diffraction peaks of ß-PVDF and the β-Ga_2_O_3_ component is shown in Figure 2a. Compared to the FTO glass substrate, the peaks at positions of 18.87°, 30.49°, 31.79°, 42.38°, and 58.15° corresponded to the (−201), (110), (002), (311), and (−313) crystal planes of β-Ga_2_O_3_, according to the standard XRD profile (JCPDS file No. 41-1103) [63]. In addition, the presence of polymer film was assigned to the peak at 20.16°, corresponding to the (110) of ß-phase PVDF [65]. Therefore, β-Ga_2_O_3_ was successfully embedded in the β-PVDF substrate. Figure 2b exhibits the morphology of β-Ga_2_O_3_. The presence of β-Ga_2_O_3_ is readily apparent as nanorods with a regular shape and homogeneous size, resembling a nanocolumn with a quadrilateral cross-section (width 500 nm and length 2 μm). Furthermore, the β-Ga_2_O_3_ nanorods are nanocrystalline, with more defects on the surface instead of a rigid form, consistent with previous studies, and the defect sizes are approximately 10~15 nm. The existence of multiple defects in single-size Ga_2_O_3_ is expected to hinder the recombination efficiency due to photogenerated electrons being trapped in the region of the defects [36,66,67]. Because the Ga_2_O_3_ film is amorphous, a variety of defects, including interface states, may serve as effective trap states at the interface to encourage trap-assisted tunneling and ultimately lead to the formation of ohmic contact with the bottom electrode. Therefore, the photogenerated electrons are expected to pass through the tunneling, forming isotropic currents and contributing to the improvement of device performance [55]. Energy-dispersive X-ray (EDX) evaluation was employed to confirm the existence of the Ga_2_O_3_ form, with a suitable composition ratio between Ga (39.84%) and O (60.16%) (Figure 2c). Furthermore, Ga and O elements were uniformly distributed in the prepared sample, according to elemental mapping (Figure 2d).

**Figure 3:**
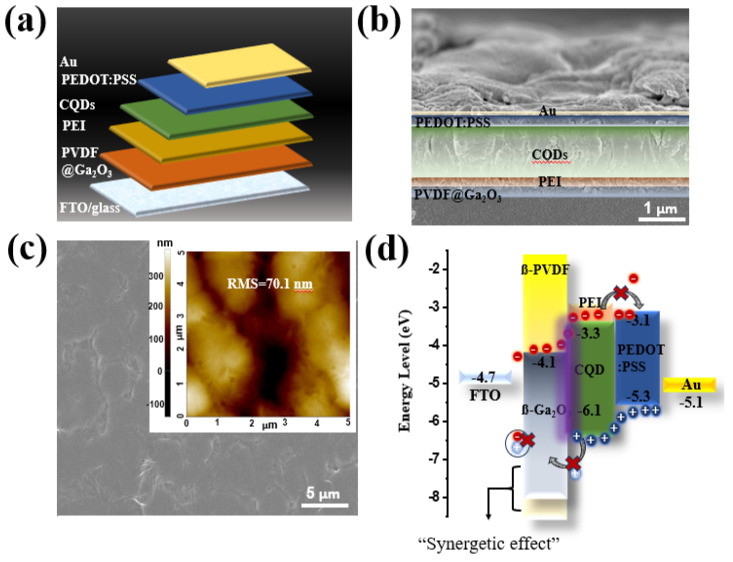
(**a**) Schematic diagram of the self-powered photodetector device; (**b**) cross-sectional SEM image of the device; (**c**) SEM image of PVDF (inset: surface morphology of spin-coated PVDF image obtained by AFM); and (**d**) operating mechanism of UVC detector under 254 nm illumination.

The structure of the self-powered PD device is shown sequentially in Figure 3a. The optical properties of the ferroelectric layer, namely ß-PVDF@ß-Ga_2_O_3_, are shown in Figure S1. ß-Ga_2_O_3_ and ß-PVDF@ß-Ga_2_O_3_ can both absorb in the visible region (Figure S1a), especially the bandgap of ß-Ga_2_O_3_ determined from the Tauc plot, measuring 4.79 eV (Figure S1b), which is slightly higher than that of previously published studies [68,69,70,71]. Additionally, the impact of the PVDF matrix on optical absorption is shown in Figure S1c. Due to its exceptional transparency (more than 90%) in the 190–250 nm wavelength, PVDF works well as a matrix for nanocomposites in UVC PDs. Therefore, ferroelectric PVDF material cannot be used as an absorber, and photo-generated electron–hole pairs can hardly be effectively separated by the induced electric field via spontaneous polarization. The Tauc profile (Figure S1d) indicated that the optical bandgap of ß-PVDF@ß-Ga_2_O_3_ was 3.85 eV, which was an appropriate energy bandgap for this research. In the cross-section SEM image in Figure 3b, the interlayers are clearly demarcated. The entire device was placed on fluorine-doped tin oxide-coated (FTO) glass. First, a 120 nm thick ferroelectric layer (comprising 40 nm PVDF and 80 nm Ga_2_O_3_) was deposited on the substrate, followed by a 60 nm PEI passivation polymer layer. The CQD photoactive layer had a thickness of ~500 nm, and the PEDOT:PSS hole transport layer had a width of 120 nm. Finally, 90 nm top electrode Au was deposited on these layers. More importantly, the uniform PVDF layer was successfully deposited on the FTO substrate with a rough surface of 70.1 nm (inset in Figure 3c). The introduction of Ga_2_O_3_ significantly reduced the surface roughness of the PVDF film (Figure S2). From EDS analysis, the uniform composition distribution of the PVDF layer with C, O, and F elements was determined (Figure S3). For the PVDF@Ga_2_O_3_ layer, the existence of inorganic filler (Ga_2_O_3_) on the PVDF surface was demonstrated (Figure S4). Therefore, the Ga_2_O_3_ acting as an inorganic filler on the PVDF matrix was successfully spin-coated despite the large size of the nanorods. The energy level distribution is shown in detail in Figure 3d, with the bandgap of the top (Au) and bottom electrode (FTO/glass) being −5.1 and 4.7 eV, respectively. In addition, the PEDOT:PSS, CQD, and Ga_2_O_3_ layers possessed energy levels of (−3.1, −5.3), (−3.3, −6.1), and (−4.1, −7.9) eV, respectively. PEDOT:PSS acted as a suitable hole-conducting layer in this study due to its higher valence band compared to the photoactive material, which contributed to attracting photogenerated holes towards the Au electrode. In our previous study, we evaluated the potential application of PEI/CQDs as photoactive materials, and, for the first time, they were used in a UVC system [15]. However, based on the band energy in Figure S5, the maximum valence band (VB) of PEI was slightly lower than that of CQD, leading to obvious hole leakage from the photoactive component to the FTO electrode, and more recombinations were formed. Thus, the PD device’s performance was limited due to the formation of an enormous dark current (Figure S6a). As such, we endeavored to form an electron transport layer with ferroelectric polymer material to increase both the performance and the applicability of the PD device. Compared to Figure S6a, we can see that the presence of Ga_2_O_3_ significantly enhanced the performance of the PD device, but the performance did not achieve high stability. The addition of Ga_2_O_3_ increased the performance of the PD device. However, the performance decreased after many cycles because only a small amount of Ga_2_O_3_ remained on the FTO substrate’s surface (Figure S6b, inset: SEM image of Ga_2_O_3_ on FTO substrate). Indeed, the small amount of Ga_2_O_3_ on the FTO surface caused inefficient photogenerated carrier transport. This is because, due to the large particle size of Ga_2_O_3_, after coating the FTO surface, these particles undergo a washout phenomenon on the substrate. In addition, the FTO surface has a high roughness without surface treatment, leading to inefficient Ga_2_O_3_ coating. Instead, the addition of PVDF formed a two-polymer system (PVDF along with PEI), which prevented the efficient transport of photogenerated electrons to the bottom electrode, leading to an unstable performance (Figure S6c). Meanwhile, the simultaneous combination of PVDF and Ga_2_O_3_ ensured stable device performance (Figure S6d). Additionally, the PD device showed high absorbance in the deep-UV region (Figure S7). The absorption peak at ~225 nm and a bump at 280 nm were attributed to the presence of photoactive CQDs. The absorption at 225 nm was assigned to the π-π* transition of the -C=C- in the sp2-hybridized domain of the graphitic core, while a bump at 280 nm was assigned to the n-π* transition of the C=O bond in the sp^3^-hybridized domains, which originated from carboxyl (–COOH) groups existing on the CQD surface [15,72,73,74]. Because of its much lower valence band compared to photoactive CQDs, Ga_2_O_3_ is an efficient electron-conducting layer that improves device performance by reducing electron–hole recombination at the FTO interface. Furthermore, the conduction band (CB) of Ga_2_O_3_ lies between the Fermi level of the FTO and that of CQDs, making it easier to extract electrons from CQDs to the FTO. Because PEDOT:PSS/CQD and Ga_2_O_3_/CQD contacts have different Schottky barrier heights, an external bias voltage is not necessary for the PD to operate. The PEDOT:PSS/CQD and Ga_2_O_3_/CQD interfaces are found to have energy barriers of roughly 0.1 and 0.8 eV, respectively. As a result, one of the primary causes of dark current is these energy barriers’ prevention of charge carriers from moving between electrodes. Electron–hole pairs are produced when the device is subjected to UV light at a wavelength of 254 nm because the photons’ energy exceeds the CQD bandgap. The lower Schottky barrier (0.1 eV) can be overcome by the photoexcited electrons, which then gather on the FTO side. The photogenerated holes simultaneously migrate to the PEDOT:PSS side for charge neutralization, producing a photocurrent at zero bias. PVDF serves as a ferroelectric layer to enhance device performance, whereas PEI is the polymer utilized in this study as a substrate to decrease trapping defects from CQDs. Furthermore, Ga_2_O_3_ functions as an efficient electron-conducting layer that increases the electronic conductivity of the PVDF passive layer while simultaneously encouraging the photogeneration of electrons from CQDs. PVDF and Ga_2_O_3_ hence have a synergistic effect.

**Figure 4:**
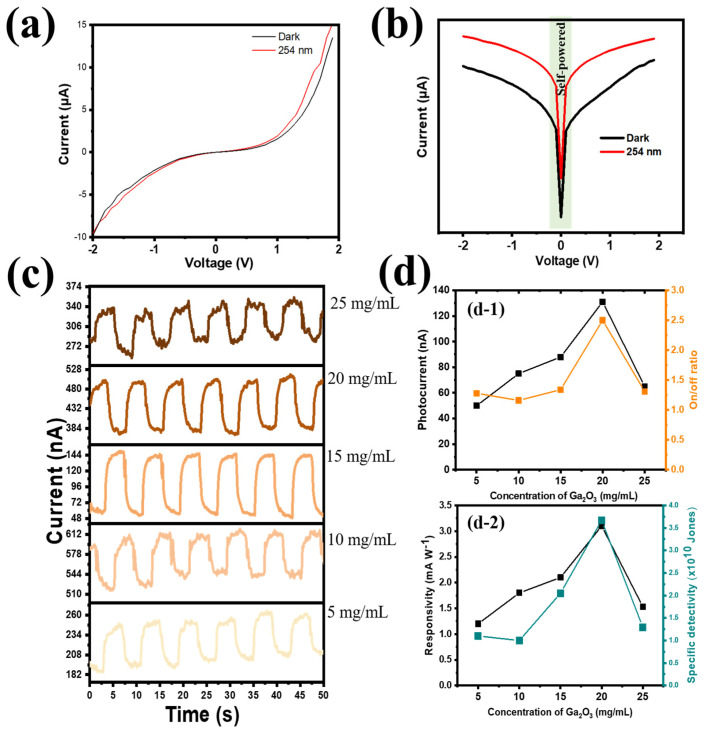
(**a**) Linear and (**b**) semi-logarithmic I–V curves of PD under dark and UVC illumination; (**c**) I-t plots under various concentrations of Ga_2_O_3_; and (**d**) performance of PD device at different concentrations of Ga_2_O_3_ with (**d-1**) photocurrent (nA) and on/off ratio and (**d-2**) responsivity (mA W^−1^) and specific detectivity (Jones).

Among the special environmental advantages of carbon-based materials are their abundance, chemical stability, low toxicity, and biocompatibility. However, the instability of CQD photoelectrons is exacerbated by their intrinsically high electrical conductivity. Because electrons trapped in intrinsic cavities and grain boundaries at the PEI/CQD interface may recombine with photogenerated holes, radiation exposure of CQDs can reduce their PD performance. Therefore, in order to limit the number of electrons trapped by the site at the CQD/PEI interface, an efficient electron transport channel was investigated in our study. Using device architecture (PEDO:TPSS/CQDs-PEI/PVDF@Ga_2_O_3_/FTO/glass), we first researched the concentration of Ga_2_O_3_ that affected the performance of PDs. The responsivity (R) of the photodetector was calculated using the following equation:

$$R=\frac{I_{photo}-I_{dark}}{PA}\;(\frac{A}{W})$$

where A is the photodetector’s active region, P is its light intensity, and I_dark_ and I_photo_ are the currents under darkness and UVC illumination, respectively. For a PD device, another important parameter is the detectivity (D*), which shows the lowest detectable light intensity of the device. D* has the following definition:

$$D^{*}=\frac{R}{\sqrt{2e\times J_{dark}}}\;(Jones)$$

where J_dark_ is the dark current density, e is the elemental charge, and R is the responsivity. It should be mentioned that D* has the following formal description: $D^{*}=\frac{{R(A)}^{0.5}{\left(\Delta f\right)}^{0.5}}{i_{noise}}$, where i_noise_ is the noise current which comes from thermal noise, shot noise, g-r noise, and 1/f noise, and Δf is the bandwidth [75,76,77]. The assumption that shot noise is a major source of noise current was employed in this work to simplify the calculation of D*. This assumption has been widely applied in earlier research on wide-bandgap material-based UV photodetectors at room temperature [75]. Our study presented the PD device’s performance in a methodical and sequential manner. From the I–V profile, the PVDF@Ga_2_O_3_-based PD showed sensitivity under UVC illumination (Figure 4a); in particular, the PD exhibited self-powered properties without applying bias voltage (Figure 4b). Next, the optimized Ga_2_O_3_ content was applied in concentrations of 25 mg mL^−1^, 20 mg mL^−1^, 15 mg mL^−1^, 10 mg mL^−1^, and 5 mg mL^−1^, marked as Ga-25, Ga-20, Ga-15, Ga-10, and Ga-5 at a fixed PVDF concentration of 10 mg/mL (PVDF-10). The photoresponse profile at different concentrations of Ga_2_O_3_ is presented in Figure 4c. Increasing the Ga_2_O_3_ concentration from 5 mg mL^−1^ to 20 mg mL^−1^, i.e., creating more carrier transport pathways, aided the tendency of photogenerated electrons to move from the CQDs to the FTO electrode. As the photocurrent, on/off ratio, responsivity, and specific detectivity were 51 nA, 1.3, 1.2 mA W^−1^, and 1.1 × 10^10^ Jones, respectively, when the lowest concentration of Ga_2_O_3_ (Ga-5) was applied, PD performance increased gradually with increasing Ga_2_O_3_ concentrations. The best concentration was achieved at Ga-20, with the highest photocurrent, on/off ratio, responsivity, and specific detectivity values of 131 nA, 2.5, 3.2 mA W^−1^, and 3.6 × 10^12^ Jones, respectively (as shown in Figure 4(d-1,d-2)). However, the excessive increase in Ga_2_O_3_ concentration disrupted the stable structure at the PVDF and Ga_2_O_3_ interface due to the large size of Ga_2_O_3_ nanorod particles, resulting in many inefficient electrons being trapped in the grain boundaries and O vacancies. Therefore, the efficiency decreased significantly with an increasing Ga_2_O_3_ concentration (25 mg mL^−1^), with the photocurrent, on/off ratio, responsivity, and specific detectivity being 65 nA, 1.4, 1.7 mA W^−1^, and 1.3 × 10^10^ Jones, respectively.

**Figure 5:**
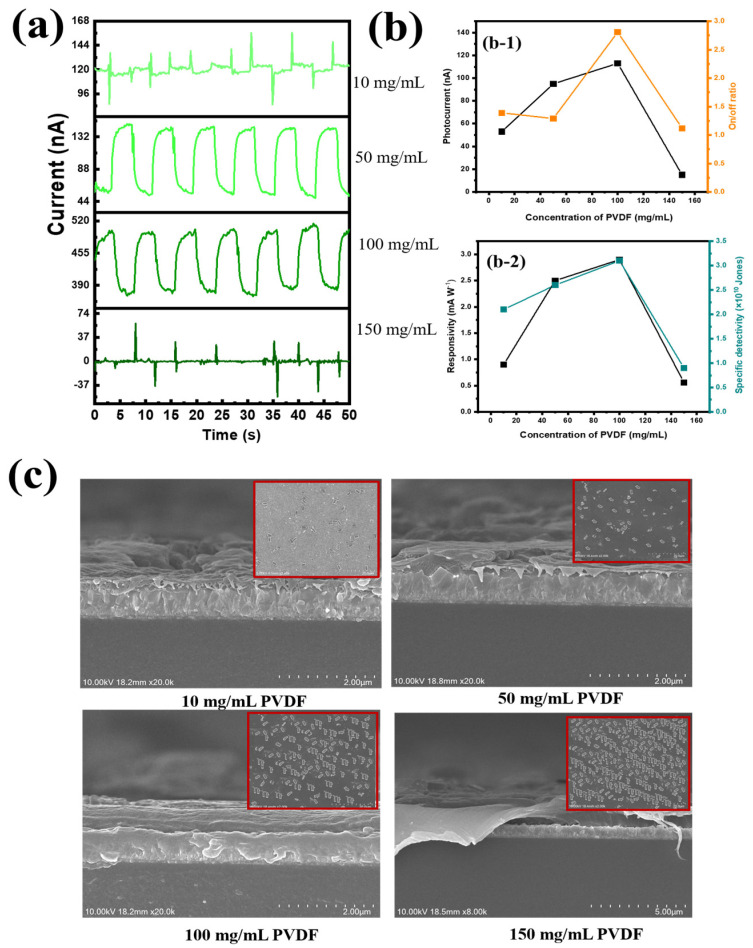
(**a**) I-t plots under various concentrations of PVDF; (**b**) performance of PD device at different concentrations of PVDF with (**b-1**) photocurrent (nA) and on/off ratio and (**b-2**) responsivity (mA W^−1^) and specific detectivity (Jones); and (**c**) cross-section SEM images at different concentrations of PVDF (inset: nanorod Ga_2_O_3_ spin-coated on PVDF substrate).

Next, the optimal content of PVDF was investigated. The polymers used in this study (PVDF and PEI) did not affect the stability of the suspension in a solution, since their zeta potentials existed at an acceptable level (−30 mV); thus, PVDF, together with PEI, was a supporting matrix for the transport of photocarriers (Figure S8). In Figure S9, we can see that the increase in PVDF content contributed to the improvement in device performance due to better signal reception under UVC illumination. In this study, the PVDF substrate served as both a supporting substrate for photogenerated electron transport and ferroelectric effects. Different PVDF contents were investigated, along with their surface morphologies, as shown in Figure 5. When we used less PVDF content (PVDF-10), the UVC signal was very weak. Due to the thick CQD and Ga_2_O_3_ layer and the enhanced electron transport routes in the absorption zone, which resulted in an unbalanced PEI/CQD and Ga_2_O_3_/PVDF interface, it was impossible for light with a wavelength of 254 nm to penetrate the PVDF layer (direction of the incident light: bottom electrode FTO → bottom PVDF@Ga_2_O_3_ → bottom PEI → CQDs → PEDOT:PSS → top electrode Au). AtPVDF-10, the photocurrent, on/off ratio, responsivity, and specific detectivity were 53 nA, 1.1, 0.9 mA W^−1^, and 2.1× 10^10^ Jones, respectively. However, the photoelectronic conductivity became more stable when the concentration of PVDF gradually increased, because the electronic conductivity would fall and the absorption of the PVDF layer would increase at this time (with the maximum absorbance peak at 255 nm and a bandgap of 3.9 eV), as depicted in Figure S1d. At a PEI concentration of 100 mg mL^−1^, the signal of the PD device grew steadily and peaked at a photocurrent of 118 nA, an on/off ratio of 2.76, a detectivity of 3.1 × 10^10^ Jones, and a responsivity of 2.6 mA W^−1^ (Figure 5(a,b-1,b-2)). Conversely, when the PEI layer thickness was beyond the 150 mg mL^−1^ threshold, the intensity of the 254 nm light that entered the absorption zone was reduced by the overly thick PEI layer. Furthermore, photogenerated electrons and holes from the photoactive CQD were unable to migrate towards the electrodes due to the PVDF@Ga_2_O_3_ film’s high PVDF content, lowering the PD device’s efficiency. Therefore, the efficiency was significantly reduced at a photocurrent of 29 nA, an on/off ratio of 1.1, a detectivity of 0.6 × 10^10^ Jones, and a responsivity of 0.7 mA W^−1^. To better understand the surface morphology changes with changing PVDF concentrations, SEM images were taken (Figure 5c). At the lowest concentration of PVDF (10 mg mL^−1^), a small amount of Ga_2_O_3_ existed on the surface. However, the Ga_2_O_3_ content gradually increased with increasing PVDF concentrations, up to the highest content of PVDF (150 mg mL^−1^). Due to the high roughness of the PVDF surface (70.1 nm), the presence of Ga_2_O_3_ acted as a drift for filler nanoparticles on the substrate, both reducing the surface tension of PVDF and enhancing network mobility for carrier transport due to the nature of PVDF as a hard polymer. However, an excessive increase in PVDF content caused fracture, i.e., the stiffness of PVDF exceeded the contact with the FTO/glass substrate, significantly reducing the device’s performance. First, because of the larger size of Ga_2_O_3_ nanorod particles, we decided to sequentially use PVDF and Ga_2_O_3_ as coatings, instead of forming a composite as in other studies. Although it is difficult to form a uniform and dense layer this way, our initial purpose was to make Ga_2_O_3_ act as an inorganic filler, supporting electron transport. Therefore, the presence of Ga_2_O_3_ on the PVDF substrate contributed to the improvement in device performance. Therefore, the concentrations of PVDF (100 mg mL^−1^) and Ga_2_O_3_ (20 mg mL^−1^) were optimal in this study.

**Figure 6:**
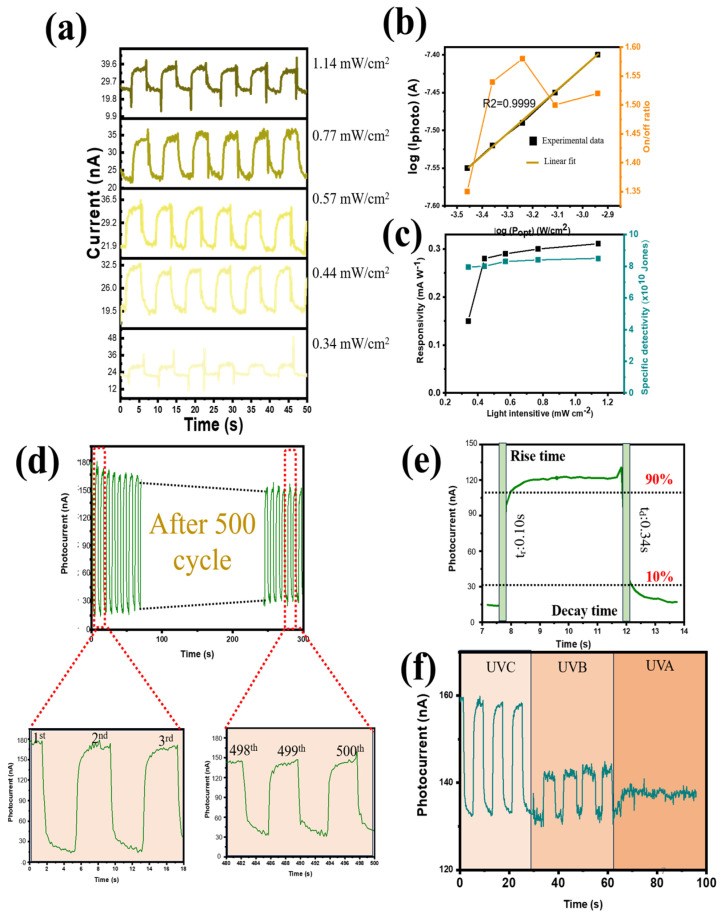
(**a**) Photocurrent under different light intensities at zero bias; (**b**) logarithm of photocurrent and on/off ratio as a function of power; (**c**) R and D* of the PD as functions of different light intensities; (**d**) long-term performance (500 cycles) when the PD was subjected to repeated on/off cycles (4 s of illumination per cycle); (**e**) rise and decay times of the PD; and (**f**) photocurrent versus time for the PD under UVC, UVB, and UVA illumination with an intensity of 1.14 mW cm^−2^.

To examine I_P_-P dependence, I-t graphs of the device were measured under self-powering conditions and 254 nm UV light in the 0.34–1.14 mW range (Figure 6a). The PVDF@Ga_2_O_3_’s augmentation of photogenerated holes and electrons caused IL to progressively rise with an increase in P, without significantly altering ID. As a result, the on/off and IP ratios were improved (Figure 6b). Furthermore, a power law fit the I_P_–P relationship: IP ∝ Pγ. In theory, a unity exponent should have been attained when all photogenerated electrons and holes were guided towards the two electrodes without recombination. However, because there were so many energy-mismatched PVDF@Ga_2_O_3_ interfaces throughout the photoactive PVDF@Ga_2_O_3_ layer, charge trapping caused the recombination of photogenerated electrons and holes at the PVDF@Ga_2_O_3_ interfaces, which was inevitable (especially at a high power intensity), meaning that γ = 0.68. Additionally, at the maximum light intensity (1.14 mW cm^−2^), the PD device performed best at a responsivity of 0.31 mA W^−1^ and a specific detectivity of 9 × 10^10^ Jones (Figure 6c). The photocurrent showed a slight degradation after 500s of irradiation, with the capacity retention of the photocurrent reaching 81% of the initial value, showing that the PD performed consistently and dependably throughout the given period of time, as shown in Figure 6d (4 s of illumination for each pulse). In addition, the photocurrent attenuation efficiency was also studied. This investigation was conducted after 500 on/off cycles (Figure S10). There was a sudden decrease in the first 500 cycles (~33 min), with capacity retention reaching 81% due to the heterostructure between the polymer and the inorganic material ETL (PVDF@Ga_2_O_3_), indicating that it took time for the interface to stabilize with the photogenerated carriers. However, the photocurrent did not decrease significantly after 7 days, with capacity retention reaching 71% under ambient air (as shown in Figure S10a), indicating good stability of the device for practical applications. In addition, as the storage time increased (under investigation conditions from 1 to 7 days), the photocurrent trend of the PD device decreased linearly (coefficient of determination (R^2^) = 0.9977) (Figure S10b). At zero bias, a high detectivity of 3.8 × 10^10^ Jones was noted because of the low dark current density. As a result, self-powered operation may enhance responsiveness and detectivity. The response time (R_t_) indicates the rise (time needed for the maximum current to increase from 10% to 90%) or decay (R_d_) (time needed for the maximum current to fall from 90% to 10%) period. Figure 6e illustrates the rise and decay times of the heterojunction PD subjected to 4 s light pulses, measuring 0.10 and 0.34 s, respectively. When exposed to irradiation at 254 nm, the PVDF@Ga_2_O_3_-based PD showed higher efficiency than when exposed to UVB and UVA light (poor signal) (Figure 6f). To demonstrate performance under self-powered conditions, a photoresponse test at reverse and forward bias voltage was conducted. The performance of the PD device was superior at 0 V compared to negative and positive piezoelectric conditions in terms of photocurrent and responsivity (Figure S11a,b). As demonstrated by its association with both positive bias voltage and responsivity, the D* and on/off ratio showed a similar tendency (Figure S11c).

**Figure 7:**
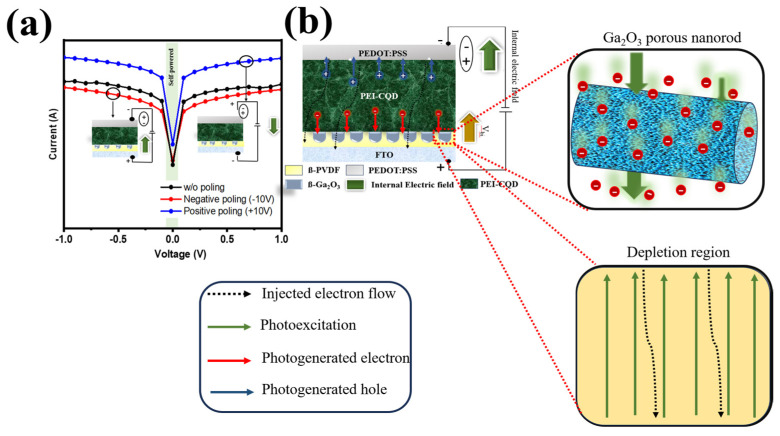
(**a**) I–V characteristics of the photodetector at +10, −10, and 0 V bias voltage under UV light illumination of 1.14 µW cm^−2^, and (**b**) schematic diagram of carrier transport dynamics induced by the electric field from negative ferroelectric materials. A + 10 V bias voltage is applied on the Au side.

**Figure 8:**
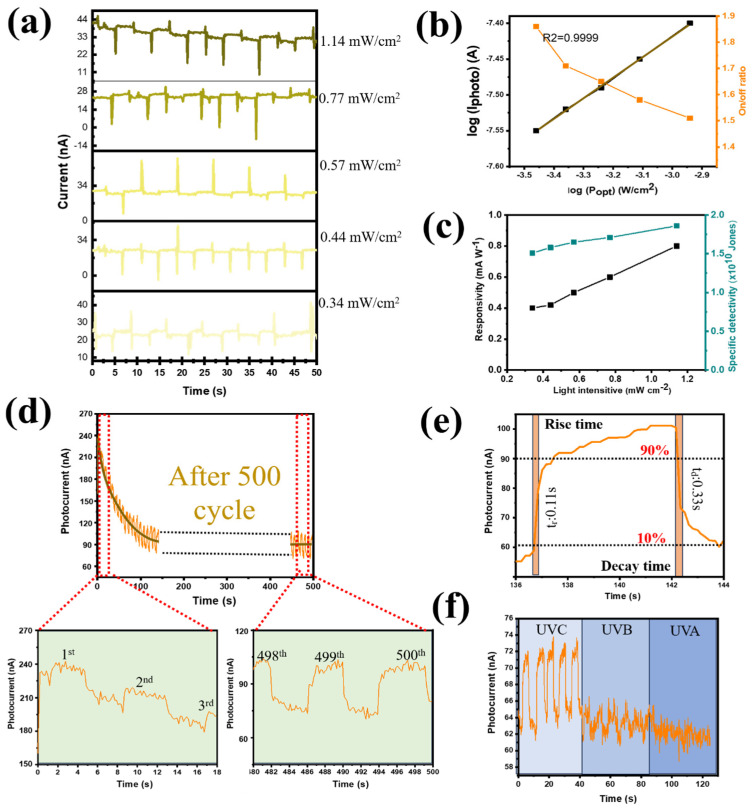
Performance of the self-powered PD with a polarized voltage of +10 V: (**a**) photocurrent under different light intensities at zero bias; (**b**) logarithm of the photocurrent and on/off ratio as a function of power; (**c**) R and D* of the PD as functions of different light intensities; (**d**) long-term performance (500 cycles) when the PD is subjected to repeated on/off cycles (4 s of illumination per cycle); (**e**) rise and decay times of the PD; and (**f**) photocurrent versus time for PD under UVC, UVB, and UVA illumination with an intensity of 1.14 mW cm^−2^.

Due to its ferroelectricity, which allows it to alternate between various photodetector operating currents, PVDF was used to modify the charge transport behavior of a self-powered photodetector. The device had features and mechanisms which could be switched depending on the polarity generated by the PVDF; these are depicted in Figure 7. When fully polarized with an applied voltage of ±10 V, the PVDF@Ga_2_O_3_-based PD device was measured under 1.14 µW cm^−2^. Different PVDF polarizations, which were regulated by varying the applied voltages, would result in varying photocurrent levels, as seen in Figure 7a and Figure S12. From the I–V profile, the PD device showed a significant increase in optical current when there was a positive bias. The enhanced drift of Ga_2_O_3_ particles on the PVDF surface together with the accumulation of photogenerated charges contributed to the PD device still receiving signals in the UVC region, even under conditions of large polarization (+10 V). Therefore, adding a ferroelectric layer (PVDF@Ga_2_O_3_) further enhanced the applicability of the PD device, especially for organic photoactive materials. In addition, the contribution of PVDF@Ga_2_O_3_ to the ferroelectric properties of the PD device was investigated (Figure S13). There was virtually no ferroelectricity for the device without the presence of PVDF@Ga_2_O_3_ (Figure S13a), which was demonstrated by the stable performance regardless of polarization changes. The application of individual PVDF or Ga_2_O_3_ components showed a non-linear change in the device’s performance (Figure S13b,c). However, the application of the PVDF@Ga_2_O_3_ composite increased the device’s performance linearly, with the polarization at +10 V showing the highest performance (Figure S13d). In Figure 7b, the working mechanism of the polarized photodetector is displayed. In summary, a dark current was created when the PD components came into contact with one another. This happened when the traps in the photoactive region allowed electrons to be pumped from the PEDOT:PSS electrode to the FTO/glass electrode. An internal electric field was created in the depletion area of PVDF@Ga_2_O_3_ after it was polarized by positive biases. This electric field lowered the dark current by blocking the injection of electrons from PEDOT:PSS to FTO. On the contrary, a favorable internal electric field aided the injection of electrons after the PVDF@Ga_2_O_3_ had been polarized by reverse biases, leading to an enhancement in the dark current. To improve the performance of the prepared UV PD, a forward bias was chosen, and the results are depicted in Figure 8.

The ferroelectric characteristics of photoactive CQDs were not extensively studied because, more importantly, the thick CQD layer had been anticipated to have multiple trapping sites at the PEI/CQD interface. Polarization facilitated charge transfer and increased the number of trapped electrons at the interface traps. However, with the special structure of Ga_2_O_3_ (nanorods with a porous structure), the photogenerated electrons were promoted for transport to the electrode due to the formation of many carrier transport pathways at the PVDF@Ga_2_O_3_ interface, minimizing the amount of trapped electrons. Therefore, the PD device based on photoactive CQDs still showed ferroelectric properties in this study. The photoelectronic performance of the self-powered UV PD is displayed in Figure 8, following the first application of a reverse voltage of +10 V. We noticed that the performance of the UVC PD was still satisfactory due to the in situ piezoelectric action of β-PVDF. When the illumination intensity was varied from 0.34 to 1.14 mW cm^−2^, the generated current had a similar trend to that of the non-polarized device (0 V polarization) as the performance increased with increasing light intensity (Figure 8a,b). The performance of the device after polarization at +10 V was achieved at an on/off ratio of 1.82, which was not much different from the unpolarized value of 2.42 (Figure 4d). In addition, the R and D* values of the self-powered UV PD were shown to be as high as 0.90 mW A^−1^ and 3.5 × 10^10^ Jones, respectively (Figure 8c). Furthermore, the repeatability and photocurrent stability of the PD device after polarization at +10 V were also improved. Figure 8d shows that the photocurrent, after decreasing in the initial 30 cycles, achieved stability in the subsequent cycles. Under forward bias in dark conditions, the contact barrier at the CQDs and PEDOT:PSS reduced, facilitating the injected electrons’ movement from the FTO to the Au electrode (Figure S14a). However, the existence of two insulating polymer systems (PVDF and PEI) effectively hindered this electron flow, keeping the dark current from being too large. Upon UVC illumination, due to the ultradeep valence band (VB) of Ga_2_O_3_ when in contact with PEI@CQD, the VB barriers did not match at the Ga_2_O_3_ interface and acted as hole-trapping sites. The holes generated in the CQD VB tended to accumulate at the hole traps, leading to further bending of the CQD conduction band (CB). Therefore, a portion of the photogenerated electrons leaked into the PEDOT:PSS layer. At the same time, the remaining photogenerated electrons recombined with photogenerated holes owing to the trapping sites at the CQD/PEI interface (Figure S14b). Therefore, the photocurrent decreased significantly from the initial cycles. However, later on, the presence of holes at the hole-trapping sites gradually decreased due to recombination, facilitating the increase in the impedance of the CQD CB, which helped to prevent the leakage of photoelectrons (Figure S14c). Therefore, after several cycles, the photocurrent of the device gradually stabilized. After 500 on/off cycles, the photocurrent capacity retention reached 83%. In addition, the PD aging effect was tested during prolonged dark storage. The PVDF and PEI polymer bilayer still effectively hindered the injected electrons’ transfer from FTO to Au, stabilizing the device, although there were slight fluctuations in the dark and light current intensities. After 7 days, the photocurrent capacity retention reached 70% (Figure S15). When a reverse bias was applied, an external electric field motivated injected electrons to move from the Au electrode to CQD@PEI, but the injected electrons still remained at a low level owing to the Schottky barrier of CQD/PEDOT:PSS junction (Figure S14d). Upon illumination, under reverse bias, the CQD/PEDOT:PSS contact barrier increased, facilitating the movement of photogenerated electrons from the CQD to the FTO electrode. However, the increase in the energy barrier contributed to the formation of electron-trapping sites at the CQD/PEDOT:PSS interface, together with hole-trapping sites at the Ga_2_O_3_@CQD interface, which significantly enhanced the accumulation of photogenerated carriers (Figure S14e,f). Therefore, the efficiency of carrier transport to charge was reduced. After several cycles, more trapping sites were created due to the increase in photogenerated electrons, leading to the increase in recombination at the CQD/PEI interface. Therefore, under reverse bias, the sensitivity of the device was not detected. In the I–V profile shown in Figure S13d, the sensitivity of the device decreases from forward bias to reverse bias. When a 254 nm UVC lamp was used, the self-powered UV PD’s response time was 0.11/0.33 s (t_rise_/t_fall_). More importantly, after polarization, the device still showed selectivity in the UVC region compared to UVB and UVA, thus ensuring the absorption properties in the deep-UV region even when applying positive voltage.

The PD described in this study competed with other CQD-based UVC PDs recently reported in terms of responsivity (3.72 mA W^−1^) and detectivity (2.35 × 10^11^ Jones) at 254 nm during self-powered operation. A previous work by Huy et al. showed response and detection capacities of 1.5 mA W^−1^ and of 1.8 × 10^10^, respectively, without ferroelectric properties [15]. With the addition of the PVDF ferroelectric polymer layer and Ga_2_O_3_ acting as an electron transport layer, our study showed better response and detectability (3.72 mA W^−1^ and 2.35 × 10^10^). More importantly, this study, for the first time, showed a PD device exhibiting ferroelectric field properties alongside good performance, including responsivity of 0.9 mA W^−1^ and detection of 3.5 × 10^10^ Jones after polarization at +10 V, and the self-powering capability of the device was another advantage in this study. In conclusion, our work suggests a practical method to maximize the usability of organic material-based PDs for a range of applications. Our findings show a straightforward and effective method for producing high-performance electrodes for optoelectronics, particularly deep-UV photodetectors.

## 4. Conclusions

In this study, we used a straightforward spin-coating method at 60 °C to successfully construct an effective UV PD based on PVDF@Ga_2_O_3_. A ferroelectric composite layer was prepared using β-Ga_2_O_3_ as a filler, and the β-phase of PVDF was used as the polymer matrix. The nanocolumn Ga_2_O_3_ content and the β-phase of PVDF were optimized to be 20 mg mL^−1^ and 100 mg mL^−1^, respectively, to form an efficient ferroelectric layer. With zero bias, the UV PD demonstrated good photoresponse characteristics to UV light at 254 nm. When the depletion zone experienced β-PVDF’s in situ piezoelectricity, the device’s performance was satisfactory. The self-powered PD exhibited specificity of D* = 3.5 × 10^10^ Jones, an on/off ratio of 2.7, and a response speed of 0.11/0.33 ms. Additionally, under continuous UV light, the produced PD demonstrated photoresponse stability, with the photocurrent achieving an 83% regeneration efficiency after about 500 s of irradiation. Our results point to a novel strategy for creating reasonably priced UV PDs for optoelectronic use in associated domains.

## Data Availability

The authors do not have permission to share data.

## Supplementary Materials

The following supporting information can be downloaded at https://www.mdpi.com/article/10.3390/nano14231903/s1.
