# Supplementary material for: Self-Powered Deep-Ultraviolet Photodetector Driven by Combined Piezoelectric/Ferroelectric Effects
Source: Nanomaterials (Basel). 2024 Nov 27;14(23):1903. doi: 10.3390/nano14231903 (PMC11642938; doi:10.3390/nano14231903)
Supplement: Supplementary file 1 [file nanomaterials-14-01903-s001.zip › nanomaterials-3323462-supplementary.pdf]

Supporting Information

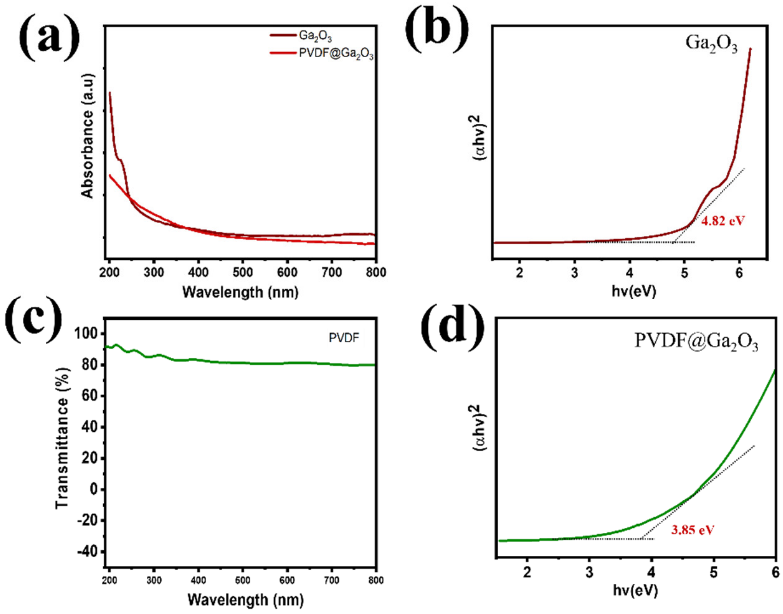

Figure S1. (a) Absorbance of Ga_2O_3 and PVDF@Ga_2O_3, (b) Tauc plot of Ga_2O_3, (c) Tranmittance of PVDF, and (d) Tauc plot of PVDF@Ga_2O_3

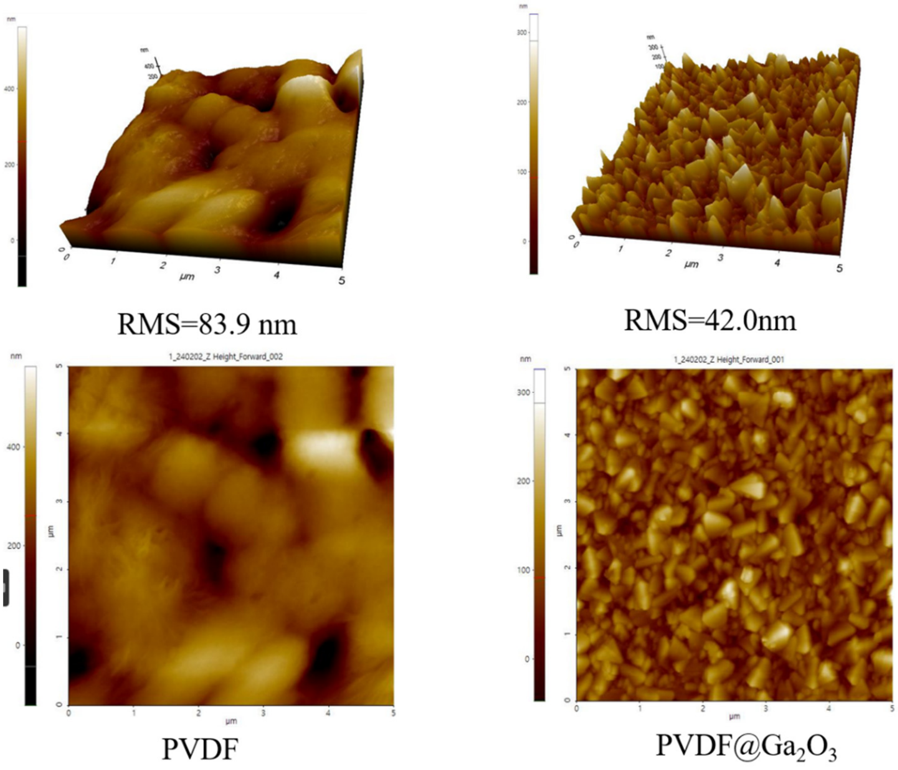

Figure S2. AFM image of PVDF and PVDF@Ga_2O_3

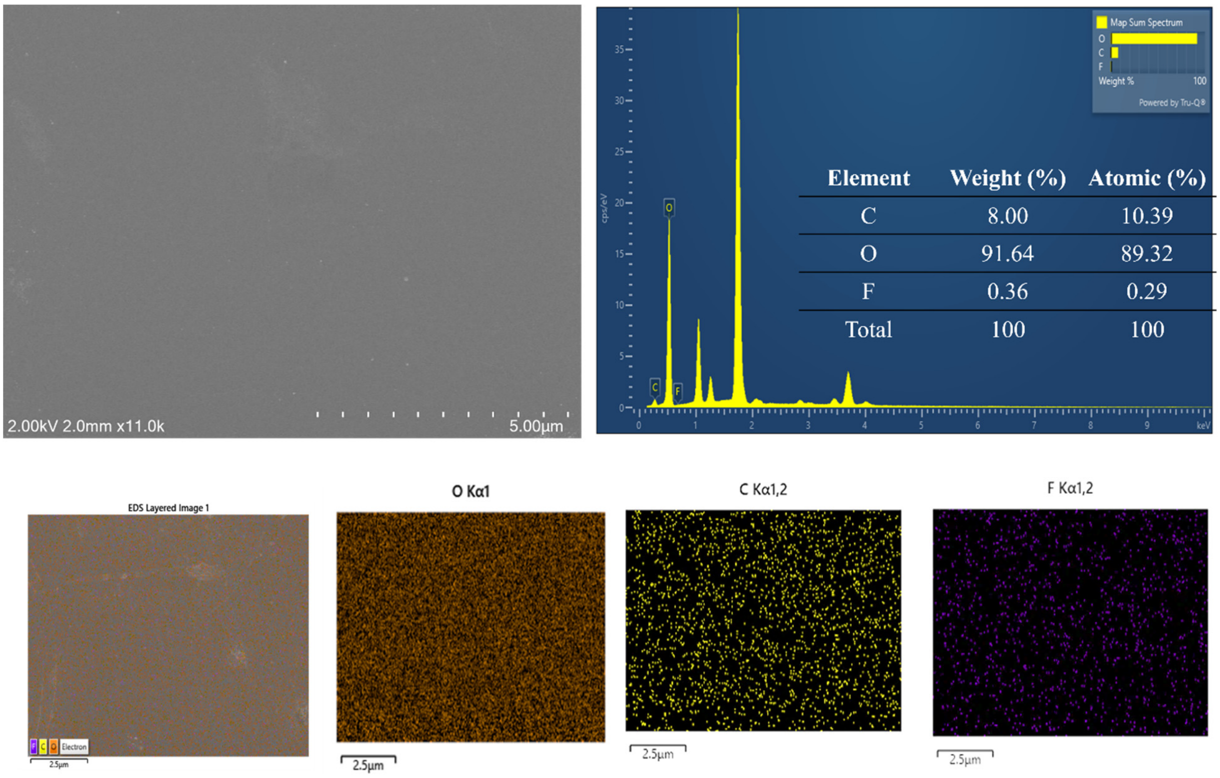

Figure S3. EDX analysis and elemental mapping of PVDF matrix

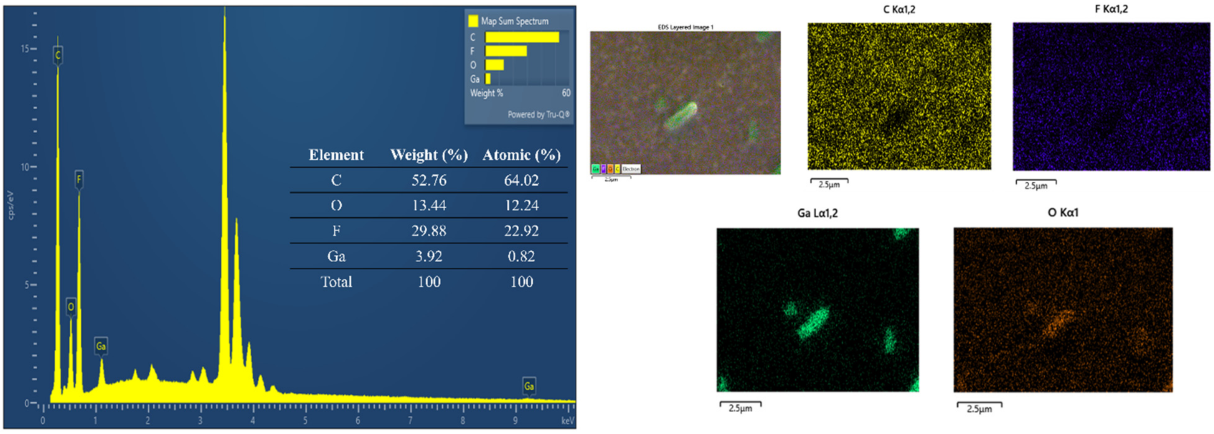

Figure S4. EDX analysis and elemental mapping of PVDF@Ga_2O_3 matrix

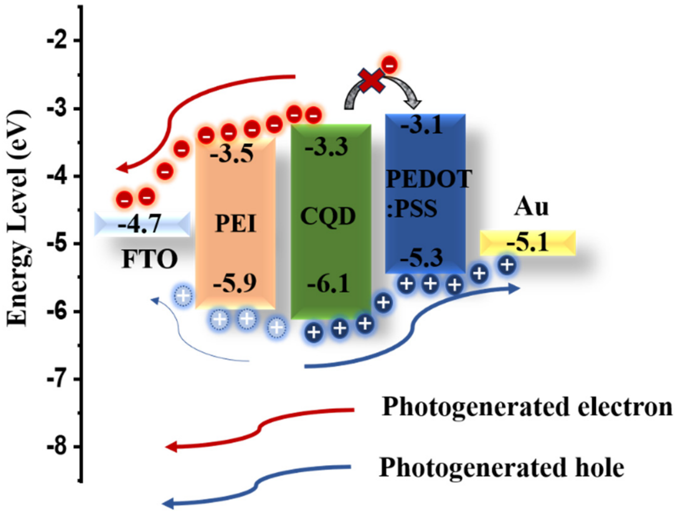

Figure S5. Band diagram of PD device (FTO/PEI/CQD/PEDOT:PSS/Au)

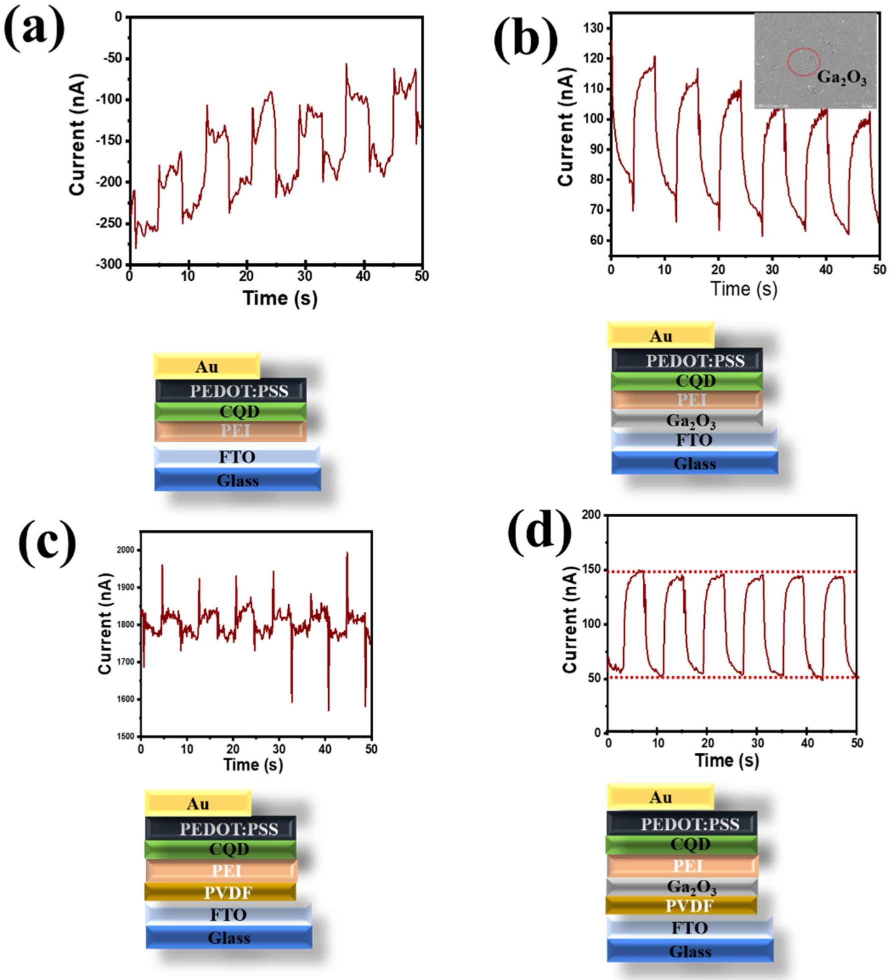

Figure S6. Photoresponse of diffent PD devices.

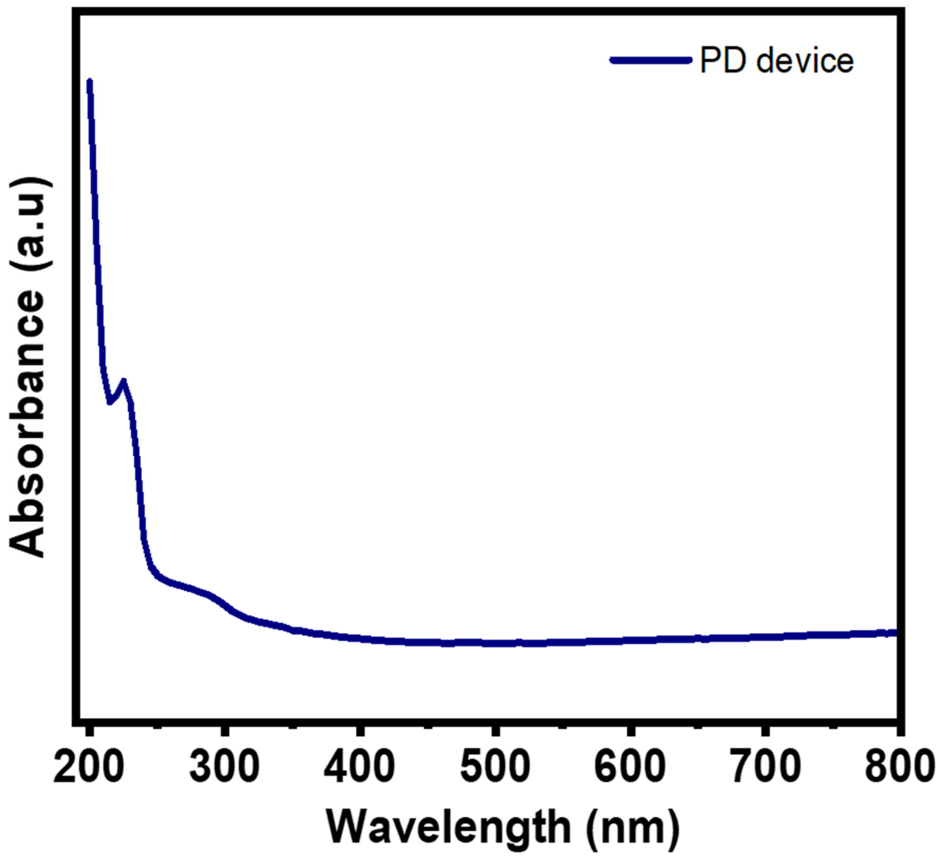

Figure S7. Absorbance of PD device

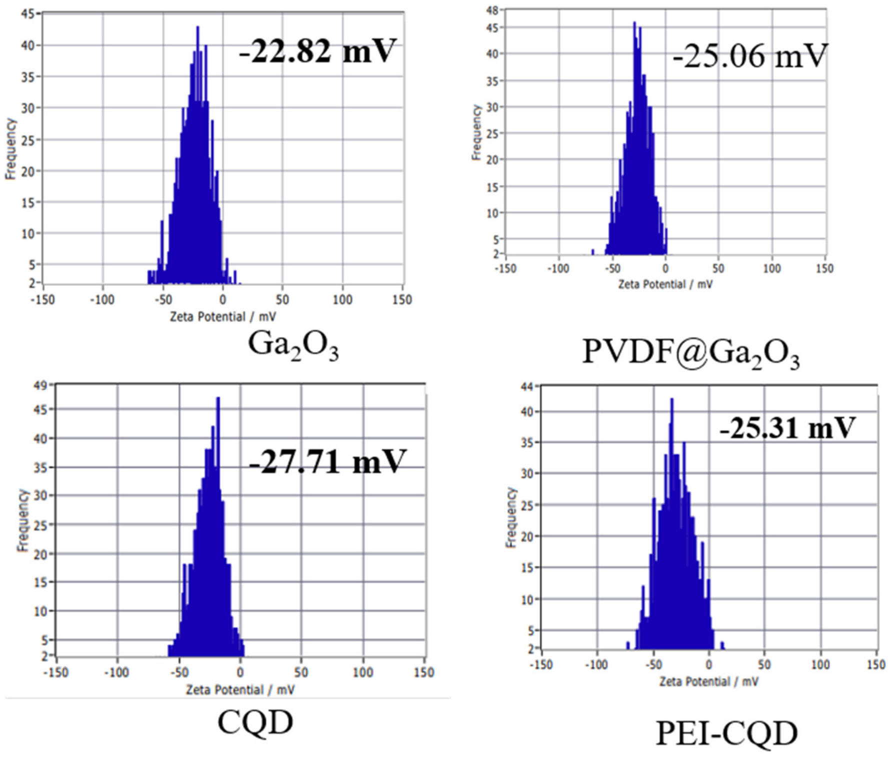

Figure S8. Zeta potential of Ga_2O_3, PVDF@Ga_2O_3, CQD, and PEI@CQD.

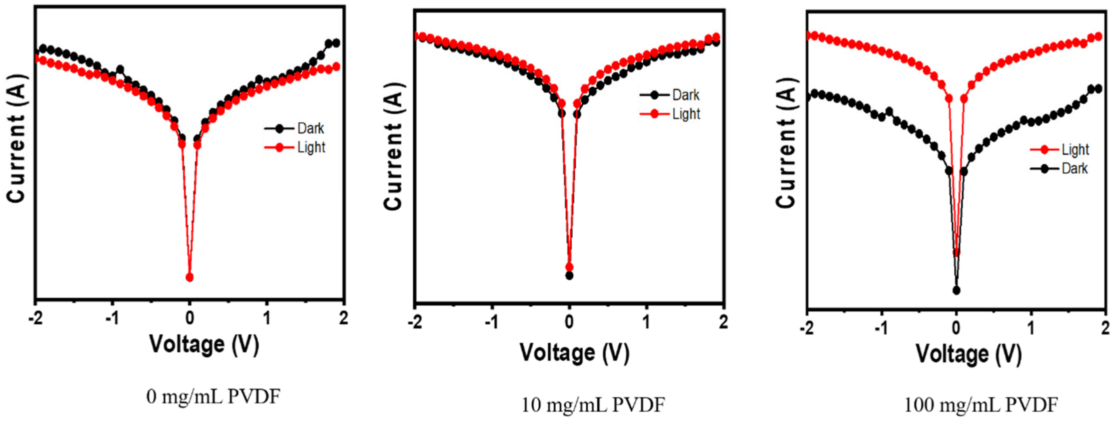

Figure S9. semi-logarithmic I–V curves of PD under dark and UVC illumination at different content of PVDF

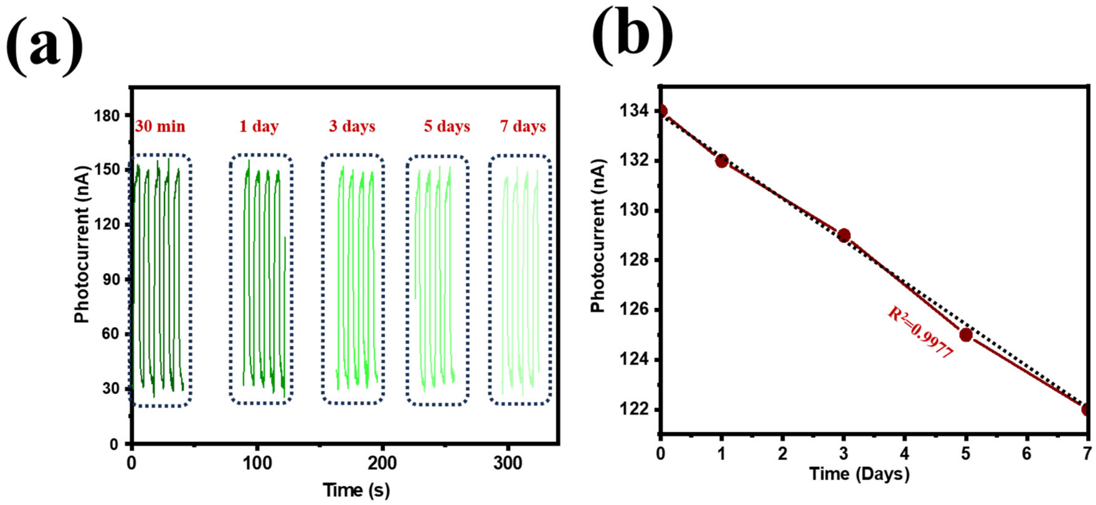

Figure S10. (a) Photoresponse of the PD device is stored in 7 days, and (b) photocurrent changed as a function of time.

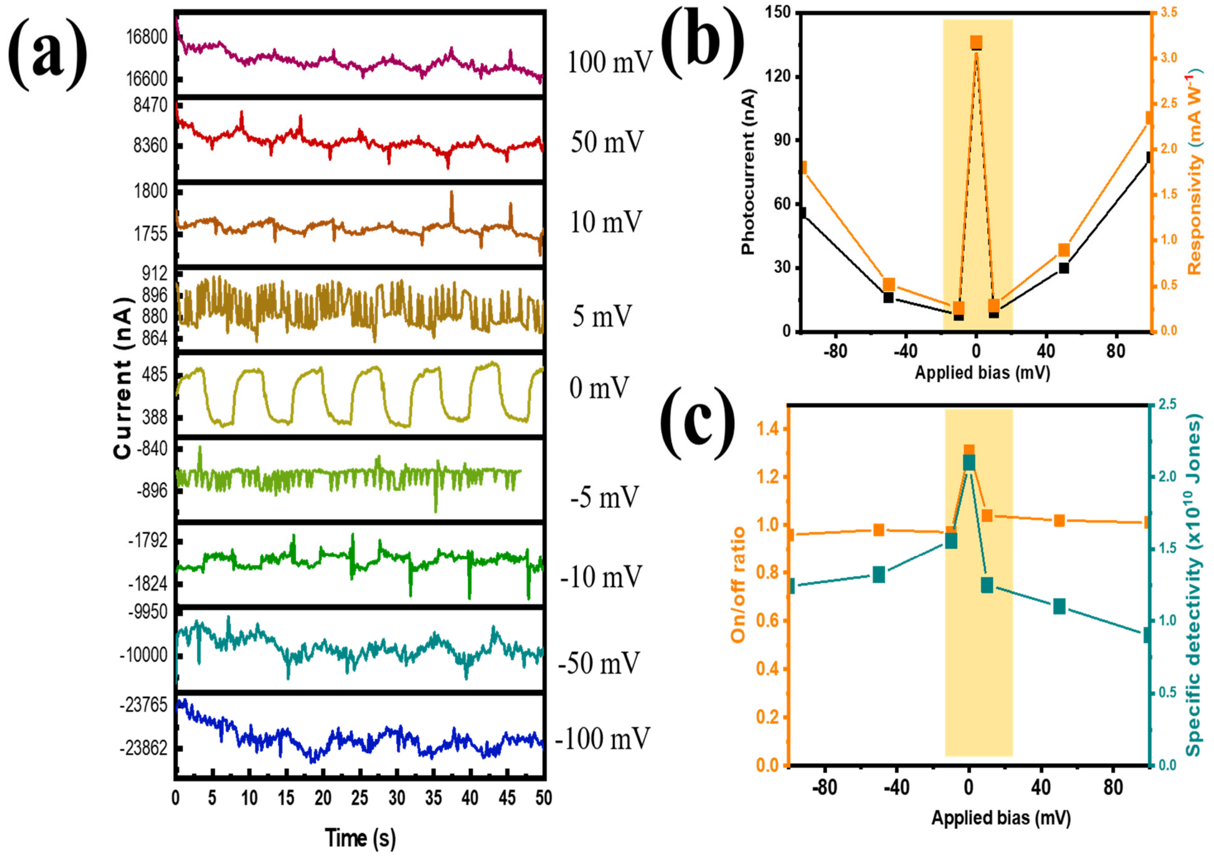

Figure S11. (a) Photoresponse, (b) Photocurrent and Responsibility, and (c) On/off ratio and specific detectivity of PD device at different bias voltage.

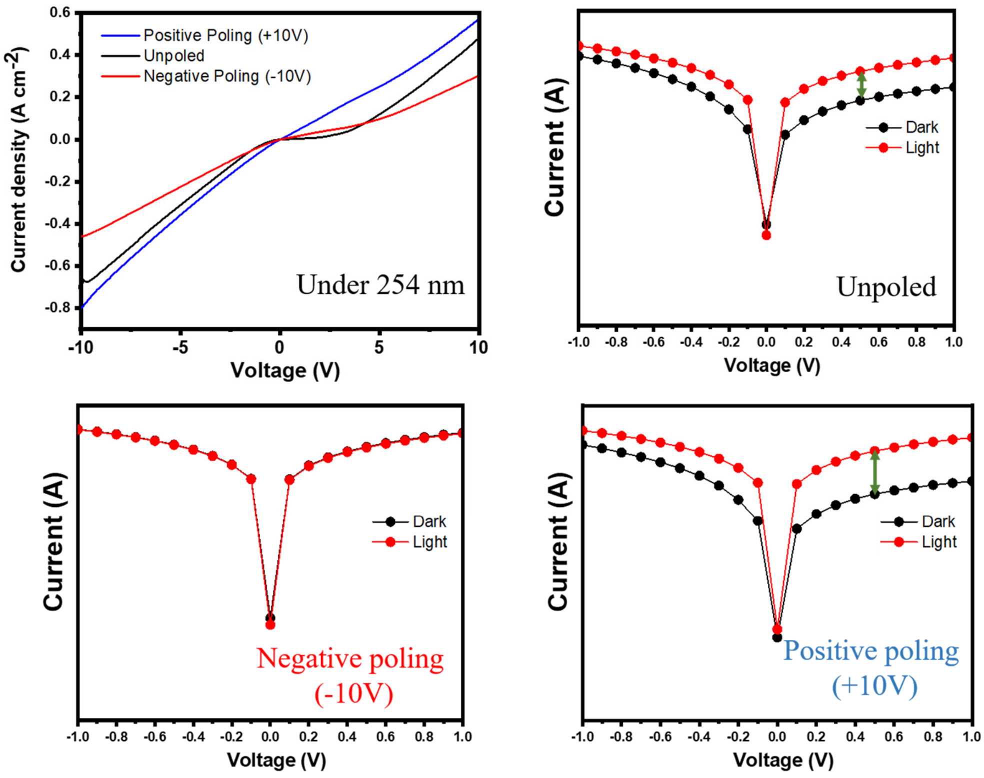

Figure S12. (a) Linear and (b) semi-logarithmic I–V curves of PD under dark and UVC illumination at different poling voltage.

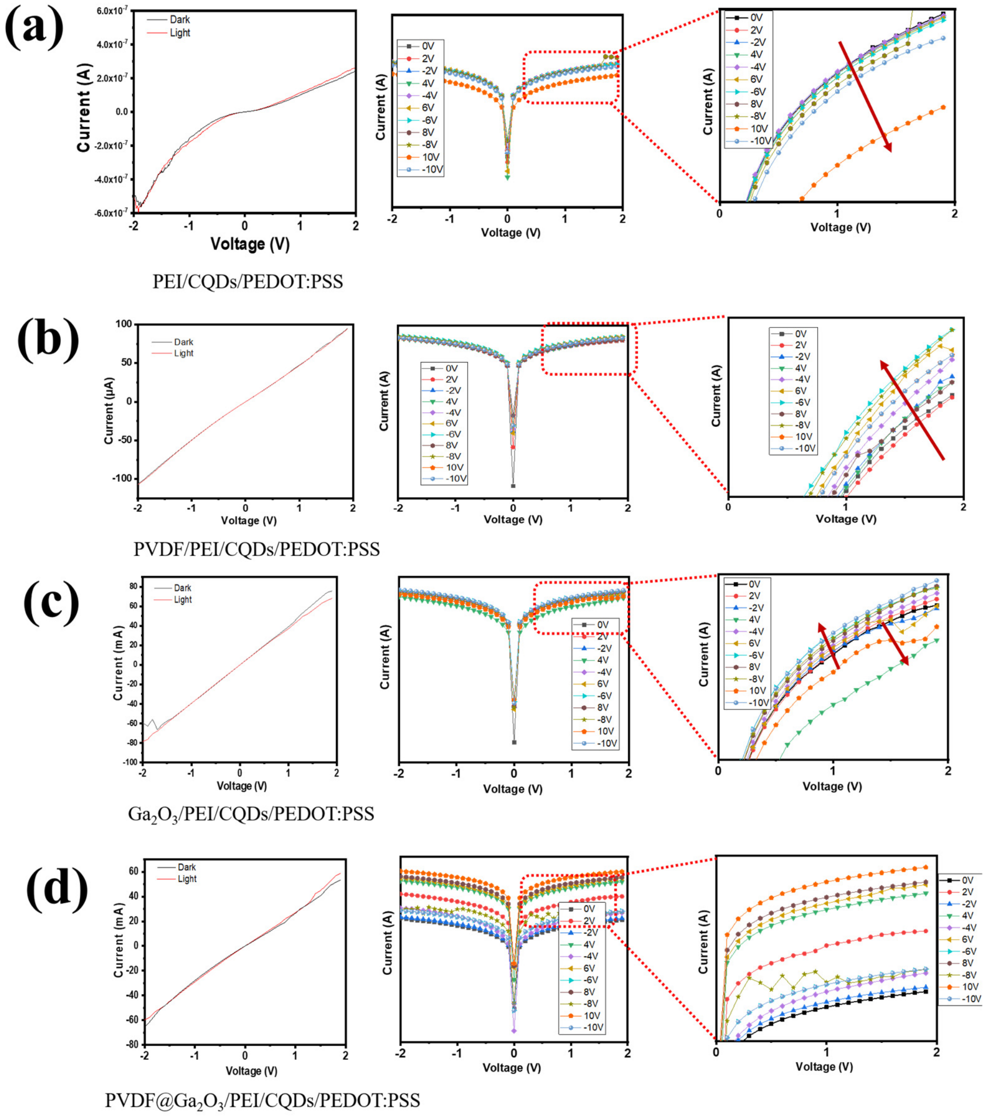

Figure S13. Linear and semi-logarithmic I–V curves of PD under dark and UVC illumination at different poling voltage at (a) PEI/CQDs/PEDOT:PSS, (b) PVDF/PEI/CQDs/PEDOT:PSS, (c) Ga_2O_3/PEI/CQDs/PEDOT:PSS, and (d) PVDF@Ga_2O_3/PEI/CQDs/PEDOT:PSS

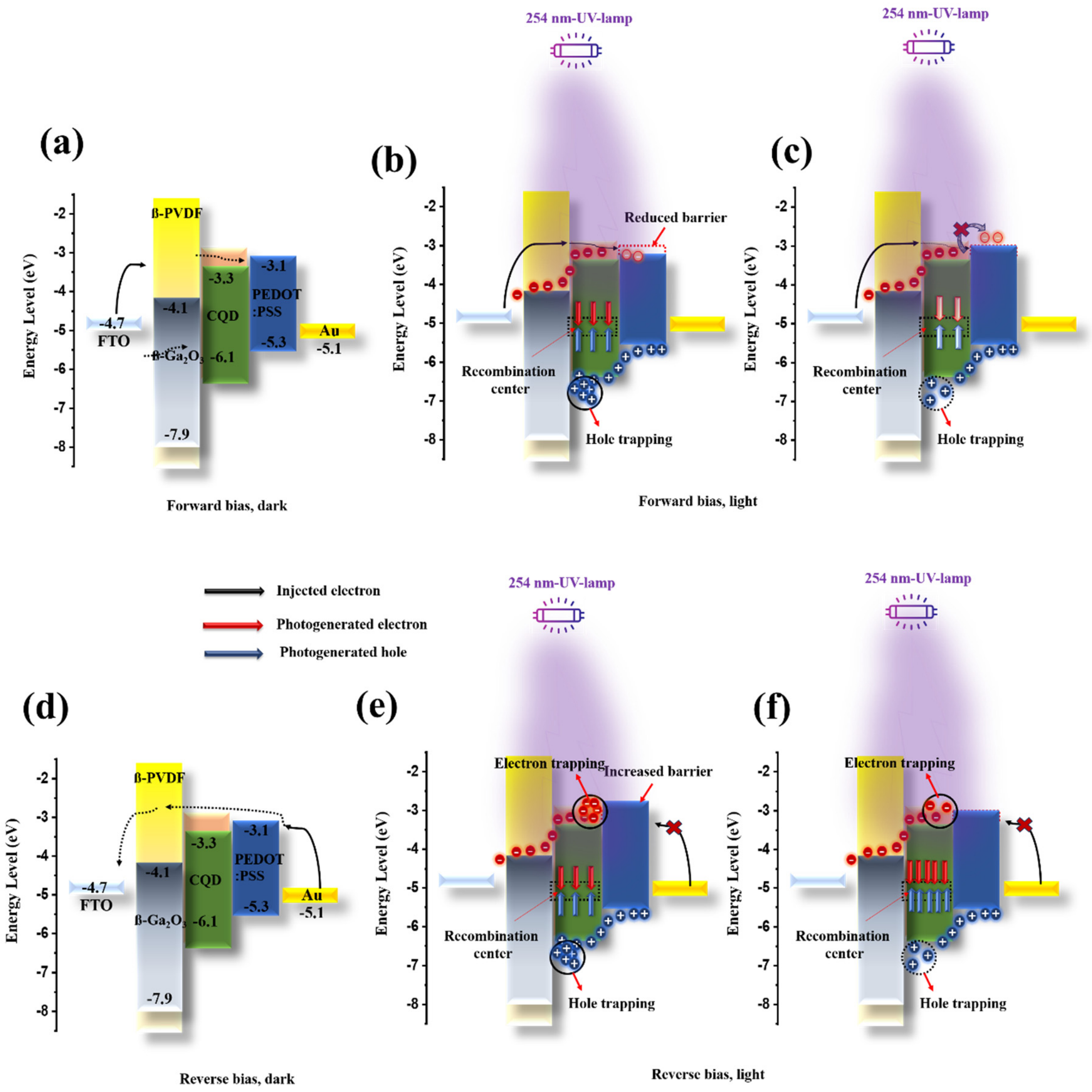

Figure S14 . Working mehcanism of PVDF@Ga$_2$O$_3$ at (a) dark conditions and (b-c) UVC light illumination under forward bias, and (d) dark conditions and (e-f) UVC light illumination under reverse bias

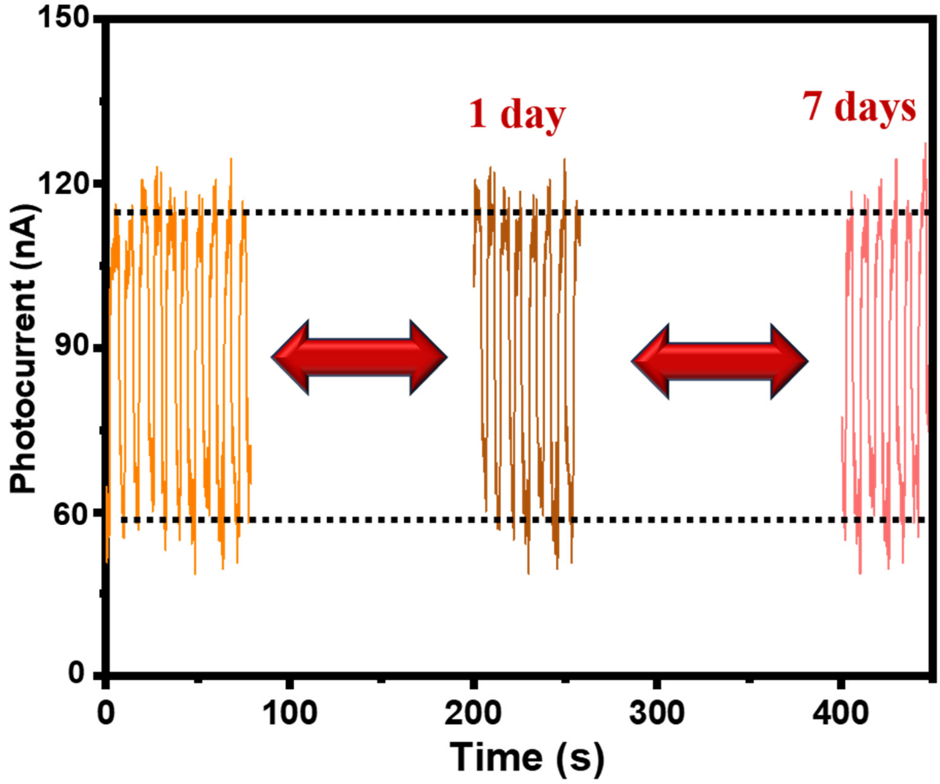

Figure S15. Photorespon of PD device under forward bias (+ 10V) after several days in the after a prolonged storage in dark condition.
